# Cardioprotection of Repeated Remote Ischemic Conditioning in Patients With ST-Segment Elevation Myocardial Infarction

**DOI:** 10.3389/fcvm.2022.899302

**Published:** 2022-05-26

**Authors:** Shaomin Chen, Shijia Li, Xinheng Feng, Guisong Wang

**Affiliations:** ^1^Department of Cardiology and Institute of Vascular Medicine, Peking University Third Hospital, Beijing, China; ^2^Key Laboratory of Cardiovascular Molecular Biology and Regulatory Peptides, Ministry of Health, Beijing, China; ^3^Key Laboratory of Molecular Cardiovascular Science, Ministry of Education, Beijing, China; ^4^Beijing Key Laboratory of Cardiovascular Receptors Research, Beijing, China; ^5^Department of Internal Medicine, Beijing Huairou Hospital, Beijing, China

**Keywords:** ST-segment elevation myocardial infarction, repeated remote ischemic conditioning, speckle-tracking echocardiography, cardioprotection, global longitudinal strain (GLS)

## Abstract

**Background:**

Repeated remote ischemic conditioning (RIC) after myocardial infarction (MI) has been shown to improve left ventricular (LV) remodeling in the experimental studies, but its cardioprotective effect in patients with ST-segment elevation myocardial infarction (STEMI) is still unknown.

**Objective:**

To investigate whether repeated RIC started early after primary percutaneous coronary intervention (PCI) can improve LV function in patients with STEMI.

**Methods:**

Patients with STEMI treated by primary PCI were included and randomized to the repeated RIC group (*n* = 30) or the control group (*n* = 30). RIC was started within 24 h after PCI and repeated daily for 1 week, using an Auto RIC device. 3D speckle-tracking echocardiography (STE) was used to assessed LV function. The primary study endpoint was the change in LV global longitudinal strain (GLS) from baseline to 1 month after PCI.

**Results:**

The repeated RIC group and the control group were well-matched at baseline including mean GLS (−9.8 ± 2.6% vs. −10.1 ± 2.5%, *P* = 0.62). Despite there was no significant difference in mean GLS at 1 month between the two groups (−11.9 ± 2.1% vs. −10.9 ± 2.7%, *P* = 0.13), the mean change in GLS from baseline to 1 month was significantly higher in the treatment group than in the control group (−2.1 ± 2.5% vs. −0.8 ± 2.3%, *P* = 0.04). There were no significant differences in the changes in global circumferential strain (GCS), global area strain (GAS), global radial strain (GRS), LV ejection fraction (LVEF), LV end-diastolic volume (LVEDV), and LV end-systolic volume (LVESV) between the two groups. Peak creatine kinase isoenzyme-MB, peak high-sensitivity troponin T, and plasma N-terminal pro-brain natriuretic peptide (NT-proBNP) levels at 24 h after PCI did not differ significantly between the two groups, but NT-proBNP levels at 1 week were significantly lower in the treatment group than in the control group [357.5 (184.8–762.8) vs. 465.0 (305.8–1525.8) pg/ml, *P* = 0.04].

**Conclusion:**

Daily repeated RIC started within 24 h after PCI can improve GLS and reduce plasma NT proBNP levels in patients with STEMI.

## Introduction

Despite the increasing use of percutaneous coronary intervention (PCI), the morbidity and mortality from acute myocardial infarction (MI) remain substantial. Therefore, adjunct cardioprotection in addition to rapid reperfusion is still needed (1). Remote ischemic conditioning (RIC) is a non-invasive procedure in which repeated brief episodes of ischemia/reperfusion (I/R) at a site distant from the heart protect the heart from IR injury (1). In the clinical setting, RIC can be performed as several cycles of 5 min ischemia followed by 5 min reperfusion using a pneumatic cuff placed on the upper arm or thigh (2). Although the mechanisms by which RIC protects the heart have not been elucidated fully, a number of clinical studies have shown that RIC performed after the onset of ST-segment elevation myocardial infarction (STEMI) could reduce infarct size and increase myocardial salvage (2–4). Several randomized controlled trial (RCT) studies showed that RIC might also improve clinical outcomes in patients with STEMI (5, 6). However, in a large multicenter RCT, RIC did not improve clinical outcomes at 12 months (7). In most of the previous clinical studies, RIC was performed around percutaneous coronary intervention (PCI) and comprised three or four cycles of cuff inflation and deflation (3–7). It is still not known if this protocol is the most effective cardioprotective intervention in terms of timing and duration.

A new concept known as repeated RIC has emerged (8). Repeated RIC, also known as chronic RIC is the daily or intermittent use of RIC for several days or weeks. Experimental animal studies have demonstrated that repeated RIC applied after MI may improve left ventricular (LV) remodeling (9, 10). However, the only published clinical study did not show the beneficial effect of repeated RIC in patients with STEMI on the improvement of left ventricular ejection fraction (LVEF) (11). In this study, RIC was started on the third day after primary PCI, but acute remodeling begins very early after MI. Moreover, LVEF may not be sensitive enough to evaluate the cardioprotective effect of the repeated RIC. Thus, this study was designed to investigate whether repeated daily RIC started early after primary PCI can improve LV function in patients with STEMI. To assess LV function, 3 dimensional (3D) speckle-tracking echocardiography (STE) was used since it provides a detailed assessment of myocardial deformation, which may not be detectable with LVEF (12).

## Methods

### Trial Design and Participants

The trial was a single-center randomized clinical study performed at the Peking University Third Hospital, and was registered at http://www.chictr.org.cn (ChiCTR1900025878). It was approved by Peking University Third Hospital Medical Science Research Ethics Committee, and was performed in accordance with the Declaration of Helsinki.

The study was conducted between January 2019 and October 2019. Criteria for inclusion were: age 18–80 years, first STEMI; successful revascularized by primary PCI within 12 h after chest pain onset. Criteria for exclusion were: past history of myocardial infarction; past history of severe peripheral vascular diseases, such as arterial occlusion, venous thrombosis, etc.; cardiac arrest; cardiogenic shock, or cardiac function grade III or above (Killip classification); serious infectious diseases, bleeding diseases, coagulation dysfunction, malignant tumors, autoimmune diseases, serious liver, and kidney dysfunction, etc.

Participants were randomized according to an IBM SPSS23.0-generated randomization schedule, with a 1:1 allocation to the repeated RIC group or control group. The study personnel performing RIC protocols were not blinded to the treatment allocation, but were not involved in data collection or analysis. Conversely, the investigators collecting and analyzing the data were blinded to the group allocation.

### Procedures

Remote ischemic conditioning treatment was conducted by well-trained study personnel during hospitalization using an automated device (AutoRIC, Xuan Yi Tong, IPC-906D, China). The treatment, which comprised 4 cycles of cuff inflation for 5 min to 200 mm Hg and deflation for 5 min, was started within 24 h after primary PCI and repeated daily for 1 week. The cuff was placed on the same arm throughout the study. In the control group, the device went through the same timed cycles but with inflation only to 10 mm Hg.

Prior to PCI, patients received 300 mg aspirin, 600 mg clopidogrel or 180 mg ticagrelor orally, and standard weight-adjusted heparin intravenously. The PCI procedures were performed following guideline recommendations (13). Thrombus evacuation and administration of glycoprotein IIb/IIIa inhibitors were at the discretion of the operators.

### The 3D Echocardiography and Speckle-Tracking Analysis

The 3D echocardiography was performed in all the patients during hospitalization after PCI (baseline) and was repeated at 1 month after PCI, using a Vivid E95 (GE) device and a 4 V-D transducer. The examination was focused on the LV. A full-volume 3D dataset was acquired over 4 consecutive cardiac cycles from the apical window using multibeat 3D mode. The frame rate was higher than 25 Hz to analyze 3D myocardial deformation. Images were recorded digitally and analyzed offline using the 4D–AutoLVQ package (Echo PAC V.110.1.3, GE Healthcare). LV endocardial border was detected automatically, and was adjusted manually if needed. LV end-diastolic volume (LVEDV) and LV end-systolic volume (LVESV) were calculated, and 3D LV ejection fraction (LVEF) was provided. Subsequently, an automatic trace of the epicardial border was performed to create the region of interest required for the myocardial deformation analysis. This epicardial trace was also adjusted manually to include the entire myocardium properly (12). Then, the strain parameters were displayed as global longitudinal strain (GLS), global circumferential strain (GCS), global radial strain (GRS), and global area strain (GAS) of the LV. Echocardiographic analyses were performed by a single cardiologist blinded to all the clinical and study data.

Intra-observer and inter-observer reproducibilities of myocardial deformation measurement were assessed in 10 randomly selected patients. To test intra-observer reproducibility, echocardiographic analyses were performed twice by the same cardiologist 2 weeks apart. To test inter-observer reproducibility, echocardiographic analyses were performed again by a second cardiologist.

### Biomarkers

Serum creatine kinase isoenzyme-MB (CK–MB) and high-sensitivity troponin T (Hs–TnT) levels were measured at admission and repeatedly every 6 h after primary PCI over the next 24 h. Peak CK–MB and Hs–TnT levels were recorded. Plasma N-terminal pro-brain natriuretic peptide (NT proBNP) levels were measured 24 h and 1 week post primary PCI.

### Study Endpoints

The primary study endpoint was the change in GLS from baseline to 1 month post PCI. Secondary study endpoints included: changes in GCS, GAS, GRS, LVEF, LVEDV, and LVESV from baseline to 1 month; MI size measured by peak levels of CK–MB and Hs–TnT; NT proBNP levels at 24 h and 1 week post PCI.

### Sample Size Estimation and Statistical Analysis

According to the previous studies, the estimated increase of the absolute value of GLS from baseline to 1 month was 1.5 ± 3.4% in patients with STEMI after successful PCI (14). It was assumed that the absolute value of GLS could be improved by 1.5% above this natural recovery by RIC (i.e., 3.0% increase in the treatment group). Thus, a sample of 23 in each group was needed for an alpha level of 0.05 and 80% power using a two-sided test. To allow for a drop-out rate of 10%, we aimed to recruit 26 patients in each group or 52 patients in total.

Continuous data are expressed as mean ± SD or median (25th, 75th percentiles), and the comparison between the two groups was conducted with Student's *t*-test or Mann–Whitney test. Categorical data are expressed as numbers (percentage), and a comparison between the two groups was conducted with the chi-square test. Changes in echocardiographic variables from baseline to 1 month were calculated for each patient, and the date were confirmed to be normally distributed using the Kolmogorov–Smirnov test. Intra-observer and inter-observer reproducibility of 3D echocardiographic parameters were assessed using intraclass correlation coefficient (ICC). All the statistical analyses were conducted using SPSS 23.0.

## Results

### Study Groups

**Figure 1:**
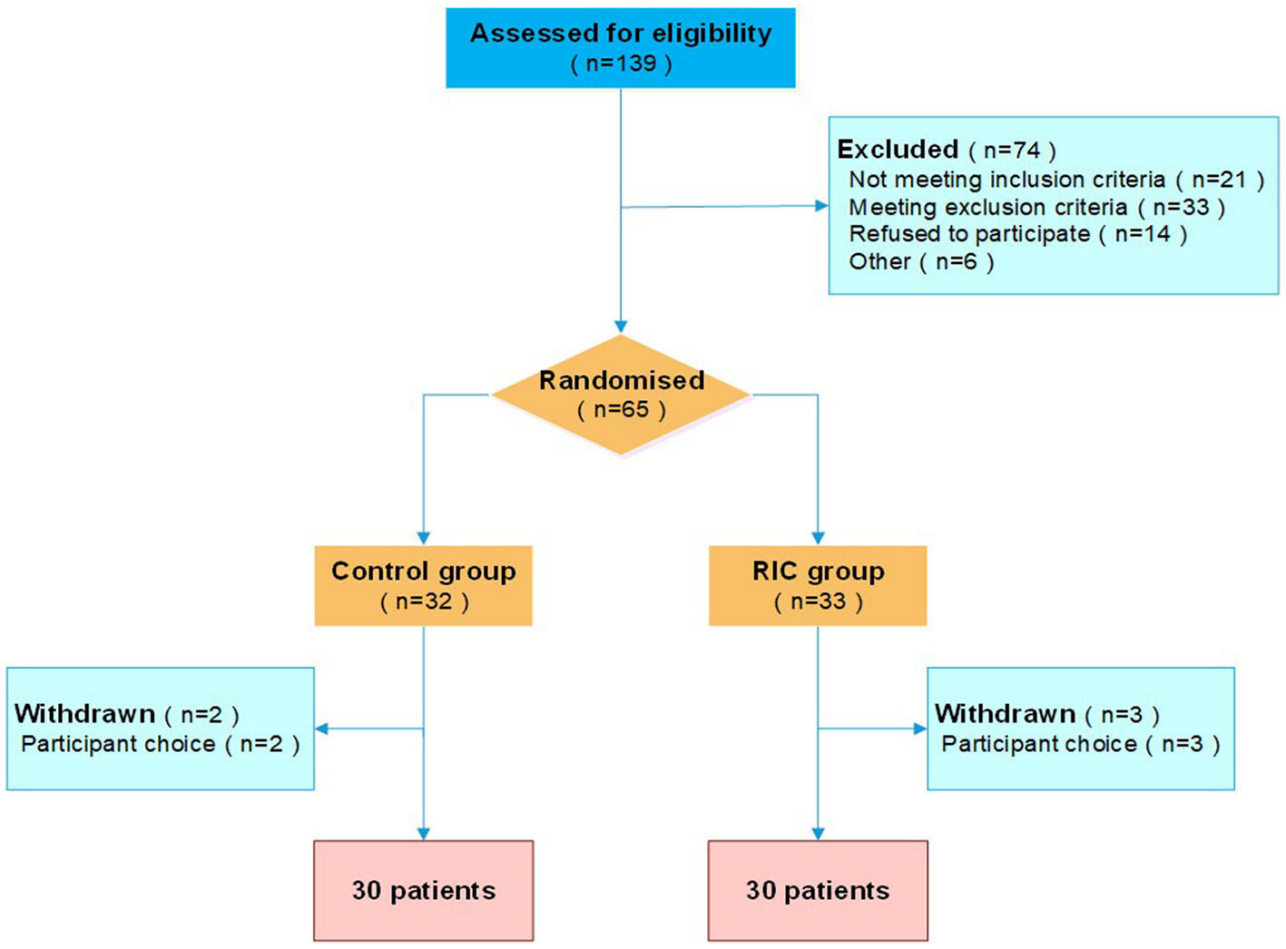
Patient flow chart. RIC, remote ischemic conditioning.

During the study period, 139 consecutive patients with STEMI undergoing primary PCI were screened for participation. Of all the eligible patients, 65 patients were randomized to the repeated RIC group or the control group. Five patients withdrew or were withdrawn after randomization and 60 patients (30 patients in the treatment group and 30 patients in the control group) completed the study (Figure 1). Participants in the treatment group and the control group were well matched in terms of characteristics and procedural data (Table 1). All the participants received drug-eluting stents, and took aspirin and a P_2_Y_12_ inhibitor thereafter.

**Table 1:** Baseline characteristics and procedural data of the RIC group and the control group.

	**RIC group**	**Control group**	***P***
	**(*n* = 30)**	**(*n* = 30)**	
Age (years)	61.7 ± 12.7	59.5 ± 12.5	0.50
Male (%)	24 (80.0%)	25 (83.3%)	0.74
BMI (kg/m^2^)	24.9 ± 2.1	25.1 ± 1.6	0.67
Diabetes mellitus (%)	8 (26.7%)	11 (36.7%)	0.41
Hypertension (%)	17 (56.7%)	17 (56.7%)	1.00
Dyslipidemia (%)	11 (36.7%)	12 (40.0%)	0.79
Current smoker (%)	18 (60.0%)	14 (46.7%)	0.30
Previous angina	13 (43.3%)	16 (53.3%)	0.44
Time from chest pain to balloon (hours)	7.3 ± 3.1	6.9 ± 3.7	0.66
Time from PCI to RIC (hours)	12.5 ± 6.9	13.9 ± 7.9	0.48
Heart rate (beats/min)	77.3 ± 12.6	81.1 ± 17.4	0.35
Systolic blood pressure (mmHg)	123.1 ± 15.5	128.8 ± 18.2	0.20
Heart function (killip's)			
I (%)	21 (70.0%)	23 (76.7%)	0.56
II (%)	9 (30.0%)	7 (23.3%)	
Culprit vessel			
LAD (%)	13 (43.3%)	13 (43.3%)	0.93
LCX (%)	5 (16.7%)	4 (13.3%)	
RCA (%)	12 (40.0%)	13 (43.3%)	
Numbers of vessels with critical stenosis			
1-vessel disease (%)	10 (33.3%)	7 (23.3%)	0.65
2-vessel disease (%)	14 (46.7%)	15 (50.0%)	
3-vessel disease (%)	6 (20.0%)	8 (26.7%)	
Initial TIMI flow grade			
0 (%)	25 (83.3%)	24 (80.0%)	0.74
I (%)	5 (16.7%)	6 (20.0%)	
TIMI flow grade after PCI			
II (%)	2 (6.7%)	1 (3.3%)	1.00
III (%)	28 (93.3%)	29 (96.7%)	
Thrombus evacuation (%)	4 (13.3%)	6 (20.0%)	0.49
Platelet GPIIb/IIIa receptor antagonist (%)	9 (30.0%)	12 (40.0%)	0.42
Gensini score	53.7 ± 20.8	50.80 ± 18.1	0.56
Length of hospital stay (days)	10.9 ± 3.4	10.0 ± 2.7	0.28
Medications after PCI			
Nitrates (%)	8 (26.7%)	10 (33.3%)	0.57
ACEi/ARB (%)	21 (70.0%)	20 (66.7%)	0.78
β blocker (%)	24 (80.0%)	22 (73.3%)	0.54
Statin (%)	30 (100%)	30 (100%)	1.00
MRA	0 (0%)	1 (3.3%)	1.00
Diuretics	15 (50%)	12 (40%)	0.44
Serum Creatinine (umol/L)	89.0 ± 42.4	83.1 ± 28.2	0.52
HGB (g/L)	148.4 ± 15.5	146.2 ± 14.9	0.56
hsCRP(mg/dl)	6.3 (1.9–30.8)	4.97 (1.7–19.8)	0.45

*Data are expressed as a mean ± SD, median (25th, 75th percentiles), or number (percentage). ACEi, ACE inhibitor; ARB, angiotensin receptor blocker; BMI, body mass index; GP, Glycoprotein; hsCRP, high-sensitivity C-reaction protein; HGB, hemoglobin; LAD, left anterior descending artery; LCX, left circumflex artery; MRA, mineralocorticoid receptor antagonist; PCI, percutaneous coronary intervention; RCA, right coronary artery; RIC, remote ischemic conditioning; TIMI, thrombolysis in myocardial infarction*.

### Echocardiographic Data

Baseline 3D echocardiography was performed at a median of 3 (1, 4) days in the treatment group and 3 (2, 4) days in the control group post PCI (*P* = 0.55). Follow-up echocardiography was performed at a median of 31 (29, 33) days in the treatment group and 32 (29, 33) days in the control group post PCI (*P* = 0.84).

**Figure 2:**
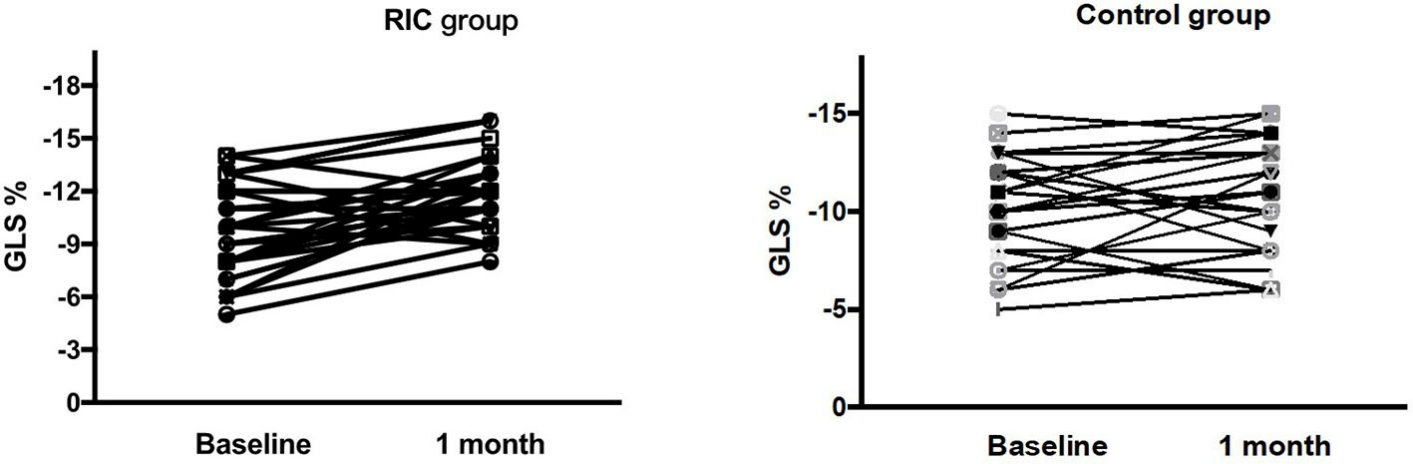
Changes of GLS from baseline to 1 month in the RIC group and the control group. GLS, global longitudinal strain; RIC, remote ischemic conditioning.

**Figure 3:**
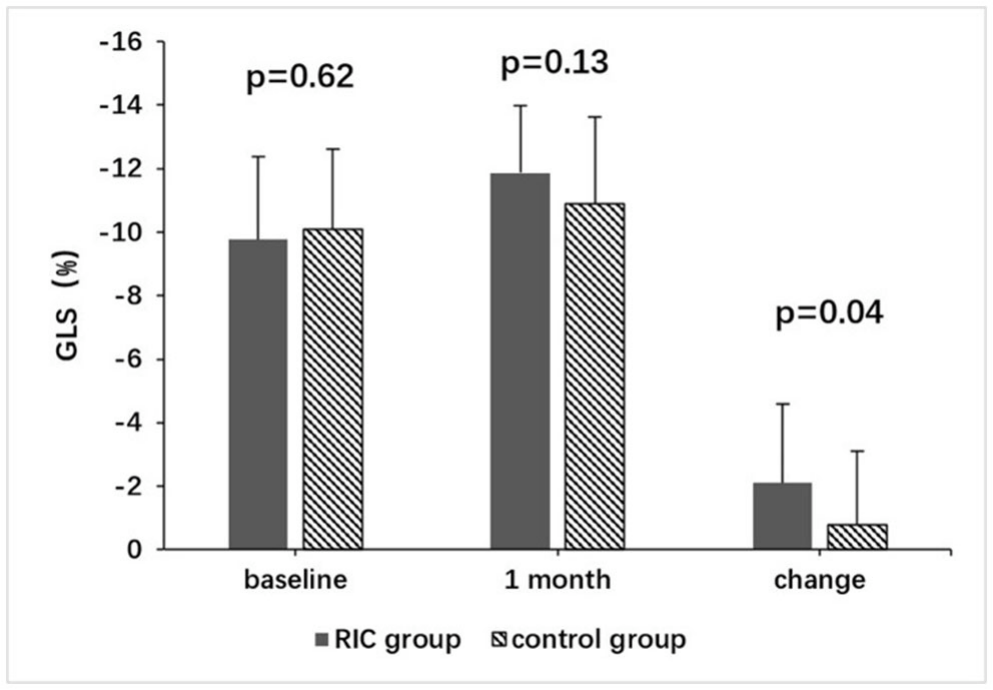
Comparison of GLS between the RIC group and the control group. GLS, global longitudinal strain; RIC, remote ischemic conditioning.

Mean GLS, GCS, GAS, GRS, LVEF, LVEDV, and LVESV at baseline were not significantly different between the two groups (Table 2). Changes of GLS from baseline to 1 month in the treatment group and the control group were shown in Figure 2. Despite there being no significant difference in mean GLS at 1 month between the two groups (−11.9 ± 2.1% vs. −10.9 ± 2.7%, *P* = 0.13), the mean change in GLS from baseline to 1 month was significantly higher in the treatment group than in the control group (−2.1 ± 2.5% vs. −0.8 ± 2.3%, *P* = 0.04) (Table 2; Figure 3).

**Table 2:** 3D echocardiographic data of the RIC group and the control group.

	**Baseline**			**1 month**			**Change**		
	**RIC group**	**Control group**	***P***	**RIC group**	**Control group**	***P*** **-values**	**RIC group**	**Control group**	***P***
**GLS (%)**	−9.8 ± 2.6	−10.1 ± 2.5	0.62	−11.9 ± 2.1	−10.9 ± 2.7	0.13	−2.1 ± 2.5	−0.8 ± 2.3	0.04
**GCS (%)**	−11.5 ± 2.8	−11.1 ± 2.4	0.59	−14.0 ± 2.5	−13.3 ± 3.4	0.35	−2.5 ± 2.4	−2.1 ± 2.8	0.59
**GAS (%)**	−18.4 ± 4.0	−17.9 ± 2.9	0.61	−19.5 ± 2.5	−19.7 ± 3.6	0.87	−1.2 ± 3.7	−1.8 ± 3.1	0.50
**GRS (%)**	26.4 ± 7.4	25.7 ± 6.3	0.72	28.8 ± 5.5	27.8 ± 7.3	0.53	2.5 ± 5.2	2.0 ± 5.8	0.76
**LVEF (%)**	51.1 ± 8.5	53.1 ± 6.9	0.31	57.2 ± 9.7	57.7 ± 8.9	0.84	6.1 ± 8.3	4.6 ± 7.2	0.45
**LVEDV (ml)**	108.4 ± 21.6	107.2 ± 21.4	0.83	112.3 ± 22.7	109.8 ± 20.4	0.66	3.9 ± 13.0	2.6 ± 11.1	0.67
**LVESV (ml)**	54.8 ± 15.4	49.6 ± 15.0	0.53	52.6 ± 12.0	47.5 ± 13.6	0.57	−5.2 ± 9.6	−5.0 ± 10.1	0.96

*Data are expressed as a mean ± SD. GAS, global area strain; GCS, global circumferential strain; GLS, global longitudinal strain; GRS, global radial strain; LVEDV, left ventricular end-diastolic volume; LVEF, left ventricular ejection fraction; LVESV, left ventricular end-systolic volume*.

With regard to secondary echocardiographic outcomes, there were no significant differences in the change in GCS, GAS, GRS, LVEF, LVEDV, and LVESV from baseline to 1 month between the two groups (Table 2).

The intra-observer and inter-observer reproducibilities for 3D echocardiographic parameters were good (Table 3). The LVEDV had the highest reproducibility, while the GRS had the lowest reproducibility.

**Table 3:** Intra-observer and inter-observer reproducibility for 3D echocardiographic parameters.

	**Intra-observer**	**Inter-observer**
GLS	0.96 (0.86–0.98)	0.93 (0.82–0.95)
GCS	0.94 (0.81–0.97)	0.93 (0.83–0.95)
GAS	0.93 (0.84–0.95)	0.90 (0.80–0.93)
GRS	0.89 (0.76–0.95)	0.87 (0.74–0.91)
LVEF	0.94 (0.79–0.98)	0.92 (0.83–0.95)
LVEDV	0.98 (0.91–0.99)	0.95 (0.89–0.97)
LVESV	0.93 (0.81–0.96)	0.91 (0.77–0.95)

*Data are expressed as intraclass correlation coefficient (ICC) with 95% CI. GAS, global area strain; GCS, global circumferential strain; GLS, global longitudinal strain; GRS, global radial strain; LVEDV, left ventricular end-diastolic volume; LVEF, left ventricular ejection fraction; LVESV, left ventricular end-systolic volume*.

### Biomarkers

Peak CK–MB and Hs—TnT values did not differ significantly between the two groups. The medium NT-proBNP level at 24 h post PCI was not significantly different between the two groups [1,053.0 (563.8–2,064.3) vs. 863.5(319.5–2,006.8) pg/ml], *P* = 0.65], but the level at 1 week when the RIC treatment was completed was significantly lower in the treatment group than in the control group [357.5 (184.8–762.8) vs. 465.0 (305.8–1525.8) pg/ml, *P* = 0.04] (Table 4).

**Table 4:** Cardiac biomarkers of the RIC group and the control group.

	**RIC group**	**Control group**	***P***
	**(*n* = 30)**	**(*n* = 30)**	
Peak CK-MB (U/L)	194.5 ± 117.1	215.3 ± 153.2	0.56
Peak Hs-TnT (ng/ml)	5.3 ± 3.1	5.8 ± 3.2	0.52
NT-proBNP at 24 h after PCI (pg/ml)	1053.0 (563.8–2064.3)	863.5 (319.5–2006.8)	0.65
NT-proBNP at 1 week after PCI (pg/ml)	357.5 (184.8–762.8)	465.0 (305.8–1525.8)	0.04

*Data are expressed as a mean ± SD or median (25th, 75th percentiles). CK–MB, creatine kinase isoenzyme-MB; Hs–TnT, high-sensitivity troponin T; NT-proBNP, N-terminal pro-brain natriuretic peptide; PCI, percutaneous coronary intervention; RIC, remote ischemic conditioning*.

## Discussion

This study has shown that repeated RIC started within 24 h post primary PCI and conducted daily for 1 week could result in a greater improvement in GLS from baseline to 1 month after PCI in patients with STEMI. There was also a significant reduction in NT-proBNP levels at 1 week. These results indicate that repeated RIC started early after PCI could improve left ventricular function in patients with STEMI treated by PCI.

A number of clinical studies have shown that RIC, as an adjunct therapy for primary PCI, could reduce the myocardial infarction size as assessed by biomarker release and cardiac magnetic resonance (MR) (2–4). Several studies also showed better clinical outcomes (5, 6). In the RIC–STEMI (remote ischemic conditioning in ST-elevation myocardial infarction as adjuvant to primary angioplasty) trial, more than 400 patients with STEMI were included, and showed that RIC reduced cardiac mortality and hospitalization for heart failure during the follow-up of more than 2 years (6). However, in the CONDI2/ERIC–PPCI (effect of remote ischemic conditioning on clinical outcomes in patients with STEMI undergoing primary PCI) trial which included more than 5,000 patients with STEMI, the implementation of RIC did not reduce infarct size (as measured by troponin release over 48 h) or the major composite endpoint events of the cardiac death and hospitalization for heart failure at 12 months (7). In this study, a complete set of troponin data were available only for <15% of patients. More than 95% of patients included in this study were classified as Killip class 1, and the event rates at 1 year were relatively low (about 9%). These might be the possible explanations for the negative results of this study (15).

In most of the previous studies including the RIC–STEMI and the CONDI2/ERIC–PPCI study, RIC comprised three or four cycles of inflation and deflation, and was conducted around the time of primary PCI (5–7). Other protocols on the RIC have also been tested for their efficiency (9–11). In a rat model of MI, Wei et al. first reported that repeated RIC administered for 28 days, either daily or intermittently (every 3 days), had a dose-dependent beneficial effect on LV remodeling as well as long-term survival. The beneficial effects were shown to be associated with less oxidative stress and inflammation (9). Yamaguchi et al. investigated the effects of repeated RIC in the chronic phase after MI in a rat model, and showed that RIC initiated 4 weeks post MI and repeated daily for 28 days could improve LV remodeling, reduce oxidative stress, and inhibit cardiac fibrosis by upregulating antifibrotic microRNAs (10). Although experimental studies have demonstrated that repeated RIC applied after MI may improve LV remodeling, its cardioprotective effect in patients with STEMI is still unknown. Vanezis et al. reported recently that daily RIC for 4 weeks in patients with STEMI after primary PCI did not improve the LVEF measured by cardiac MR 4 months after MI (11). In this study, RIC was started on the third day after primary PCI. Acute remodeling begins very early after MI (16), which might be one of the possible explanations for the negative result of Vanezis' study. Therefore, in our study, RIC was started within 24 h after primary PCI. Furthermore, RIC was conducted by well-trained personnel during hospitalization in our study, in order to make sure the treatment was performed properly. In contrast, in the study by Vanezis et al., RIC was conducted by the patients themselves.

The most commonly used parameter of LV systolic function is LVEF measured by 2D echocardiography, 3D echocardiography, cardiac MR or single-photon emission computed tomography (SPECT) (17). Although a number of studies showed that RIC reduced infarct size and myocardial edema assessed by cardiac MR in patients with STEMI, most of them failed to show improvement of LVEF by RIC (4, 18). Thus, LVEF may not sensitive enough to evaluate the cardioprotective effect of RIC. STE provides a detailed assessment of global and regional LV myocardial deformation, which may not be detectable with LVEF. Longitudinal, circumferential, and radial strains are the three directions of myocardial deformation that can be evaluated by STE. By 3D STE, GLS, GCS, and GRS can be simultaneously acquired (12). Moreover, 3D STE also provides GAS, which combines the longitudinal and circumferential deformation and reflects the change in the myocardial area (12). Global longitudinal strain (GLS) which reflects the function of subendocardial longitudinal myofibers, has been shown to be sensitive to ischemia and increased wall stress (12). In the previous studies, GLS was proved to be a strong predictor of mortality, heart failure and cardiac death in patients with STEMI, even in the absence of reduced LVEF (19). Therefore, we used 3D STE to assess LV function in this study, and found that the change in GLS from baseline to 1 month after PCI was significantly higher in the RIC group than in the control group. However, the change of LVEF from baseline to 1 month was not significantly different between the two groups. As noted, the average LVEF of the patients with STEMI included in this study was higher than 50%, but GLS was significantly impaired, with an average level of about −10%, indicating that GLS was more sensitive than LVEF in the detection of systolic dysfunction. In a previous study by Pryds et al., daily RIC for 4 weeks in patients with the chronic heart failure could improve GLS in patients with high-plasma NT-proBNP level (20).

In this study, peak CK–MB and Hs–TnT levels were not significantly different between the two groups. This may be because RIC was performed after PCI, but not around the time of PCI, and could not reduce the infarct size. The potential benefit of repeated RIC after PCI is the improvement of myocardial function after PCI, but not the reduction of infarct size.

Previous studies have demonstrated that the NT proBNP level measured in the acute phase of STEMI correlates with infarct size, and could predict myocardial function and short-term mortality after MI (21, 22). This study showed that plasma NT proBNP level in the RIC group was significantly lower than that in the control group at 1 week post PCI, indicating a better myocardial function recovery in the RIC group.

## Limitations

In this study, despite GLS was significantly improved in repeated RIC group as compared with the control group, there were no significant improvement in LVEF as well as in LVEDV. This indicates that GLS was more sensitive than LVEF to evaluate the cardioprotective effect of RIC. However, the clinical significance is still unknown.

The sample size is relatively small. Moreover, the follow-up time is relatively short, which cannot fully reflect the cardiac remodeling after myocardial infarction, and the effects of RIC on myocardial stunning cannot be unequivocally distinguished from the effects on LV remodeling (23, 24). Therefore, it is necessary to do further studies with a larger sample size and long-term follow-up to confirm the cardioprotective effect of repeated RIC in patients with STEMI after primary PCI. The baseline 3D echocardiography was performed at a median of 3 days after PCI, but not before the implementation of RIC, since patients were in the cardiac care unit when RIC was started, and 3D echocardiography could not be done at the bedside. The patients classified as Killip classes 3 or 4 were excluded since the mortality among these patients is high, but the endpoints of this study were the changes of the echocardiographic parameters from baseline to 1 month after PCI. However, these patients may potentially benefit more from RIC (25).

This study aimed to explore the cardioprotective effect of the repeated RIC started early post PCI. Thus, we did not perform RIC around the time of PCI. The combination of RIC around the time of PCI and chronic repeated RIC could potentially provide the better cardioprotection. There are several ongoing studies testing the effect of the combination protocol (26, 27).

## Conclusions

Daily repeated RIC started within 24 h after primary PCI can improve GLS and reduce plasma NT proBNP levels in patients with STEMI post primary PCI. Therefore, it can be concluded that repeated RIC can improve myocardial function recovery after MI.

## Data Availability Statement

The raw data supporting the conclusions of this article will be made available by the authors, without undue reservation.

## Ethics Statement

The studies involving human participants were reviewed and approved by Peking University Third Hospital Medical Science Research Ethics Committee. The patients/participants provided their written informed consent to participate in this study. Written informed consent was obtained from the individual(s) for the publication of any potentially identifiable images or data included in this article.

## Author Contributions

All authors listed have made a substantial, direct, and intellectual contribution to the work and approved it for publication.

## Funding

This work was supported by the National Natural Sciences Foundation of China (81870271 to GW).

## Conflict of Interest

The authors declare that the research was conducted in the absence of any commercial or financial relationships that could be construed as a potential conflict of interest.

## Publisher's Note

All claims expressed in this article are solely those of the authors and do not necessarily represent those of their affiliated organizations, or those of the publisher, the editors and the reviewers. Any product that may be evaluated in this article, or claim that may be made by its manufacturer, is not guaranteed or endorsed by the publisher.

## References

[1] Heusch G. Myocardial ischaemia-reperfusion injury and cardioprotection in perspective. Nat Rev Cardiol. (2020) 17:773–89. 10.1038/s41569-020-0403-y 32620851

[2] Heusch G. 25 years of remote ischemic conditioning: from laboratory curiosity to clinical outcome. Basic Res Cardiol. (2018) 113:15. 10.1007/s00395-018-0673-2 29516255

[3] White SK Frohlich GM Sado DM Maestrini V Fontana M Treibel TA. Remote ischemic conditioning reduces myocardial infarct size and edema in patients with ST-segment elevation myocardial infarction. JACC Cardiovasc Interv. (2015) 8:178–88. 10.1016/j.jcin.2014.05.015 25240548

[4] Heusch G Bøtker HE Przyklenk K Redington A Yellon D. Remote ischemic conditioning. J Am Coll Cardiol. (2018) 65:177–95. 10.1016/j.jacc.2014.10.031 25593060 PMC4297315

[5] Sloth AD Schmidt MR Munk K Kharbanda R K Redington AN Schmidt M. Improved long-term clinical outcomes in patients with ST-elevation myocardial infarction undergoing remote ischaemic conditioning as an adjunct to primary percutaneous coronary intervention. Eur Heart J. (2014) 35:168–75. 10.1093/eurheartj/eht369 24031025

[6] Gaspar A Lourenço AP Pereira MÁ Azevedo P Roncon-Albuquerque R Marques J. Randomized controlled trial of remote ischaemic conditioning in ST-elevation myocardial infarction as adjuvant to primary angioplasty (RIC-STEMI). Basic Res Cardiol. (2018) 113:14. 10.1007/s00395-018-0672-3 29516192

[7] Hausenloy DJ Kharbanda RK Møller UK Ramlall M Collier L. Effect of remote ischaemic conditioning on clinical outcomes in patients with acute myocardial infarction (CONDI-2/ERIC-PPCI): a single-blind randomised controlled trial. Lancet. (2019) 394:1415–24. 10.1016/S0140-6736(19)32039-2 31500849 PMC6891239

[8] Chong J Bulluck H Fw Ho A Boisvert WA Hausenloy DJ. Chronic remote ischemic conditioning for cardiovascular protection. Cond Med. (2019) 2:164–9. 32313876 PMC7169952

[9] Wei M Xin P Li S Tao JP Li YP Li J Liu MY Li JB. Repeated remote ischemic postconditioning protects against adverse left ventricular remodeling and improves survival in a rat model of myocardial infarction. Circ Res. (2011) 108:1220–5. 10.1161/CIRCRESAHA.110.236190 21474817

[10] Yamaguchi T Izumi Y Nakamura Y Yamazaki T Shiota Y Sano S. Repeated remote ischemic conditioning attenuates left ventricular remodeling via exosome-mediated intercellular communication on chronic heart failure after myocardial infarction. Int J Cardiol. (2015) 178:239–46. 10.1016/j.ijcard.2014.10.144 25464262

[11] Vanezis AP Arnold JR Rodrigo G Lai FY Debiec R Nazir S. Daily remote ischaemic conditioning following acute myocardial infarction: a randomised controlled trial. Heart. (2018) 104:1955–62. 10.1136/heartjnl-2018-313091 29748420 PMC6252375

[12] Nabeshima Y Seo Y Takeuchi M. A review of current trends in three-dimensional analysis of left ventricular myocardial strain. Cardiovasc Ultrasound. (2020) 18:23. 10.1186/s12947-020-00204-3 32591001 PMC7320541

[13] Levine GN Bates ER Blankenship JC Bailey SR Bittl JA Cercek B. 2015 ACC/AHA/SCAI focused update on primary percutaneous coronary intervention for patients with st-elevation myocardial infarction: an update of the 2011 ACCF/AHA/SCAI guideline for percutaneous coronary intervention and the 2013 ACCF/AHA guideline for the management of ST-elevation myocardial infarction. J Am Coll Cardiol. (2016) 67:1235–50. 10.1016/j.jacc.2015.10.005 26498666

[14] Xu L Cai Z Xiong M Li Y Li G Deng Y. Efficacy of an early home-based cardiac rehabilitation program for patients after acute myocardial infarction: a three-dimensional speckle tracking echocardiography randomized trial. Medicine. (2016) 95:e5638. 10.1097/MD.0000000000005638 28033254 PMC5207550

[15] Heusch G Gersh BJ. Is cardioprotection salvageable? Circulation. (2020) 141:415–7. 10.1161/CIRCULATIONAHA.119.044176 32078426

[16] Bhatt AS Ambrosy AP Velazquez EJ. Adverse remodeling and reverse remodeling after myocardial infarction. Curr Cardiol Rep. (2017) 19:71. 10.1007/s11886-017-0876-4 28660552

[17] Sekaran NK Crowley AL de Souza FR Resende ES Rao SV. The role for cardiovascular remodeling in cardiovascular outcomes. Curr Atheroscler Rep. (2017) 19:23. 10.1007/s11883-017-0656-z 28357714

[18] Crimi G Pica S Raineri C Bramucci E De Ferrari GM Klersy C. Remote ischemic post-conditioning of the lower limb during primary percutaneous coronary intervention safely reduces enzymatic infarct size in anterior myocardial infarction: a randomized controlled trial. JACC Cardiovasc Interv. (2013) 6:1055–63. 10.1016/j.jcin.2013.05.011 24156966

[19] Iwahashi N Kirigaya J Abe T Horii M Toya N Hanajima Y. Impact of three-dimensional global longitudinal strain for patients with acute myocardial infarction. Eur Heart J Cardiovasc Imaging. (2020) 10.1093/ehjci/jeaa241 32995886

[20] Pryds K Nielsen RR Jorsal A Hansen MS Ringgaard S Refsgaard J. SchmidtMR. Effect of long-term remote ischemic conditioning in patients with chronic ischemic heart failure. Basic Res Cardiol. (2017) 112:67. 10.1007/s00395-017-0658-6 29071437

[21] Mayr A Mair J Schocke M Klug G Pedarnig K Haubner BJ. Predictive value of NT-pro BNP after acute myocardial infarction: relation with acute and chronic infarct size and myocardial function. Int J Cardiol. (2011) 147:118–23. 10.1016/j.ijcard.2009.09.537 19896736

[22] Kwon TG Bae JH Jeong MH Kim YJ Hur SH Seong IW. N-terminal pro-B-type natriuretic peptide is associated with adverse short-term clinical outcomes in patients with acute ST-elevation myocardial infarction underwent primary percutaneous coronary intervention. Int J Cardiol. (2009) 133:173–8. 10.1016/j.ijcard.2007.12.022 18281115

[23] Heusch G. Myocardial stunning and hibernation revisited. Nat Rev Cardiol. (2021) 18:522–36. 10.1038/s41569-021-00506-7 33531698

[24] Heusch G Libby P Gersh B Yellon D Böhm M Lopaschuk G. Cardiovascular remodelling in coronary artery disease and heart failure. Lancet. (2014) 383:1933–43. 10.1016/S0140-6736(14)60107-0 24831770 PMC4330973

[25] Cheskes S Koh M Turner L Heslegrave R Verbeek R Dorian P. Field implementation of remote ischemic conditioning in ST-segment-elevation myocardial infarction: the FIRST study. Can J Cardiol. (2020) 36:1278–88. 10.1016/j.cjca.2019.11.029 32305146

[26] Song L Yan HB Zhou P Zhao HJ Liu C Sheng ZX. Effect of comprehensive remote ischemic conditioning in anterior ST-elevation myocardial infarction undergoing primary percutaneous coronary intervention: design and rationale of the CORIC-MI randomized trial. Clin Cardiol. (2018) 41:997–1003. 10.1002/clc.22973 29726013 PMC6489915

[27] Zheng Y Reinhardt JD Li JN Hu DY Lin S Wang LS. Can clinical and functional outcomes be improved with an intelligent “internet plus”-based full disease cycle remote ischemic conditioning program in acute ST-elevation myocardial infarction patients undergoing percutaneous coronary intervention? rationale and design of the i-RIC trial. Cardiovasc Drugs Ther. (2022) 36:45–57. 10.1007/s10557-020-07022-9 32607820

